# Repeated implants failure in young patient with idiopathic nephrotic syndrome: a case report with brief review of the literature

**DOI:** 10.1186/s12903-023-03772-8

**Published:** 2024-01-05

**Authors:** Lamees R. Alssum

**Affiliations:** Department of Periodontics and Community Dentistry, College of Dentistry, King Saud University, Riyadh, Saudi Arabia

**Keywords:** Idiopathic nephrotic syndrome, Corticosteroids, Dental implants

## Abstract

**Background:**

Nephrotic syndrome is a chronic disorder characterized by heavy proteinuria, hypoalbuminemia, hyperlipidemia, and edema. Idiopathic minimal-change disease is the most common form encountered in children. Corticosteroids are the cornerstone for the treatment of idiopathic nephrotic syndrome (INS), with different regimens depending on the response to therapy and frequency of relapses. This case report presents complications after implant treatment in patient with INS.

**Case presentation:**

20 years old female patient presented for implant consultation. Medical history includes INS since early childhood, and she is on different medications to control her condition, including long-term steroid use. Dental history revealed that implant treatment was unsuccessful after multiple attempts. She presented with an implant on the area of lower left first mandibular molar, that shows increased mobility and radiolucency on radiographic examination. A diagnosis of implant failure was made, the implant was removed, and the area was cleaned and sutured. The patient decided to replace her missing teeth with fixed partial denture and was referred for prosthodontist. The potential adverse effect of steroid use and the possible underlying mechanism that could affect bone metabolism and implants osseointegration are reviewed.

**Conclusion:**

Clinical practice guidelines are needed for the management of dental implants in chronic steroid users.

## Introduction

Nephrotic syndrome (NS) is a chronic disorder with alterations of the permselectivity at the glomerular capillary wall, resulting in its inability to restrict the urinary loss of protein. It is characterized by heavy proteinuria, hypoalbuminemia, hyperlipidemia, and edema [1]. Idiopathic nephrotic syndrome (INS) is the most frequent form of NS in children [2]. Minimal change disease (MCD) represents cases with minor changes on kidney biopsy examination under light microscopy and accounts for 80% of cases.

The cause of INS remains unknown, but the pathogenesis is hypothesized to involve immune dysregulation, systemic circulating factors, or inherited structural abnormalities of the podocytes. Cell mediated immunity dysfunction, particularly T lymphocytes, play a role in the pathogenesis of NS [3]. Abnormalities of T cell subsets and/or their function have been variably reported in a number of patients with MCD [4, 5]. CD80 (B7-1) is a protein expressed on antigen-presenting cells that can cause T- cell activation, and recent studies identified it as the cause of podocytopathies and proteinuric states. The T-cell surface expresses protein receptor CTLA-4, which binds CD80. An increase in podocyte B7-1 expression has been evident in a variety of animal models of proteinuria and in human studies [6].

Corticosteroids are the keystone for INS treatment according to guidelines from the International Study of Kidney Disease in Children and the Arbeitsgemeinschaft für Pädiatrische Nephrologie [7, 8]. Most of the children with INS (90–95%) respond to steroid therapy [1, 3]. The response to steroid therapy and the frequency of the relapses provide a guide for therapy. Although most children have a good initial response to steroids, they have periods of relapses. Individuals with frequent relapses become steroid dependent [1–3] or require steroid-sparing agents, such as cyclophosphamide, ciclosporin, or rituximab [2, 9, 10]. This case report presents a young patient with repeated implant failures and INS. The possible underlying mechanisms that affect bone metabolism and implant osseointegration are discussed.

## Case presentation

A 20-year-old female patient came to the dental college at King Saud University, to get another consultation regarding her implant. The implant was installed at another dental center about a year prior. The patient expressed frustration in the past few years for trying to have her missing teeth replaced with dental implants, but the treatment was unsuccessful. She decided to seek treatment at another clinic.

The patient’s medical history included INS with minimal change disease (MCD) since childhood. The diagnosis was made when the patient was around 2 years old. The patient had been on steroids with various regimens and periods since then. Medical records showed that the patient is taking different medications to control her condition, including prednisolone, lisinopril, calcium, vitamin D, and iron supplements. She also received 2 courses of rituximab. She had regular follow-up appointments with her physician every few months, and according to the most recent one, the MCD in full remission, and the patient would be on a maintenance dose of rituximab at 1 g every 12 months.

The most recent bone density scan (DXA) on her medical records revealed normal bone mineral density. Her dental history included some restorations, endodontic treatments, and extractions in the few past years with no complications. The patient had 4 implants installed during the last 2 years. Records showed that 2 implants were placed at the area of the upper right first maxillary molar (tooth #16) with indirect sinus lift, and both failed before restoration (failure of osseointegration). Similarly, 2 implants were placed at the area of the lower left first mandibular molar (tooth #36), and the first one failed before the final restoration, while the second one was still present, and the patient was seeking consultation for it. On patients’ last dental visit few months ago, patient was told that the implant failed and need to be removed. However, patient did not continue treatment. Patient noticed that implant got exposed to the oral cavity with increased mobility and it has migrated into more coronal position during the last year.

Periodontal examination showed good oral hygiene, slight gingival inflammation localized on the lower anterior sextant, and a pocket depth range of 1–3 mm. The patient’s periodontal condition was diagnosed as localized plaque-induced gingivitis. The patient had a full set of dentition except for teeth #16 and 36, which were extracted due to caries. The patient had one implant in the area of tooth #36 with a cover screw. The implant was exposed to an oral cavity with soft tissue recession and showed increased mobility.

**Fig. 1:**
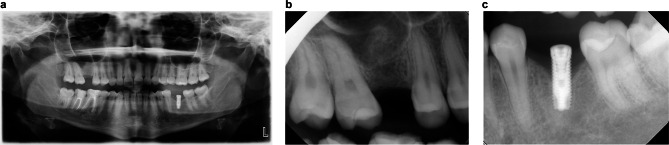
radiographs taken at the first dental visit. **a** Panoramic radiograph shows all teeth are present except teeth #16 and 36 and an implant at the area of tooth #36. **b** Bone graft at the right maxillary sinus is evident, indicative of indirect sinus lift procedure on previous implant placement attempts in area of tooth #16. **c** Implant at the area of tooth #36. Crestal bone loss is seen with thin radiolucent line around the implant body. Implant is migrated to a more coronal position as a result of loss of osseointegration

Panoramic and periapical x-rays were taken for the area of tooth #36 and #16 (Fig. 1, a-c). Implant #36 showed crestal bone loss, migrated to more coronal position, and a radiolucent line noted around the implant body (Fig. 1c). A diagnosis of implant failure (failure to osseointegrate) was made for #36. The implant was removed using dental forceps under local anesthesia. Soft tissue encapsulated the implant and the implant osteotomy site. The implant site was cleaned, degranulated, irrigated, and sutured.

Sutures were removed after 2 weeks, and the site healed uneventfully. Treatment options to replace the missing teeth presented to the patient. One option is to have comperhensive consultation with the patient’s doctor including necessary clinical, radiographic and laboratory tests and explore the possibility of placing implants following the physician recommendation. The other option is to replace the teeth with fixed partial dentures The patient chose the second option and was referred to a prosthodontist. Additional information for the patient’s previous implant experience were obtained from the patient’s dental records. All implants were placed by the same certified periodontist as a two-stage protocol (with cover screw) following the manufacturer’s recommendations. Primary stability was achieved in all implants with no intraoperative complications, and antibiotics were prescribed after each procedure. An informed consent was obtained from the patient to use non personal identifying details for this case report.

## Discussion

Dental professionals deal with medically compromised patients with various medical conditions who need implant surgery. The patient’s medical condition should be assessed thoroughly before implant placement. Several systemic diseases and conditions may affect bone quality or interfere with the normal healing process. In addition, the use of medication to control diseases may disturb the healing process of the implants and the surrounding tissues. Medical conditions such as immunosuppression, active malignancy treatment, uncontrolled diabetes mellitus, smoking, and intravenous bisphosphonates are considered contraindications for implant placement [11, 12]. This case report shows the possible effect of patient condition and medications (steroids) on the overall bone healing and implant osseointegration.

Implant success greatly depends on normal bone metabolism and turnover. Alveolar bone around the newly placed implant undergoes a series of cellular and molecular changes. This leads to formation of direct bone implant contact in a process called osseointegration [13] and results in implant stability to withstand functional load. Current understanding of the peri-implant wound healing represents the complexity of cell interactions, where the bone cells’ response is highly regulated by the immune cells in what is called the “osteoimmune system.” After implant insertion, both innate and adaptive immunity mechanisms are essential for bone healing [14]. Innate immunity regulates the initial inflammatory phase of the healing process, while adaptive immunity controls the tissue healing and regeneration phase around the newly placed implant [15, 16].

INS is a chronic condition characterized by a triad of proteinuria, hypo-albuminemia, and edema. Patients with nephrotic syndrome are at risk for life-threatening infections and thromboembolic episodes. Other complications include hypertension, hyperlipidemia, and features of steroid toxicity [17]. Steroids have been the first line of treatment for childhood nephrotic syndrome for over 60 years, and over 80–90% of patients achieve complete remission with prednisone or prednisolone treatment. With steroid therapy, mortality has fallen from 35 to 3% because of a reduction in serious infections [2]. Unfortunately, most of these patients have one or several relapses and need additional courses of steroids therapy. Treatment guidelines for the first manifestation and relapse of nephrotic syndrome are mostly standardized and based on practice guidelines rather than clinical trials [18].

Steroids are anti-inflammatory medications that are often used for the treatment of inflammatory non-infectious diseases. It is commonly used for the treatment of various acute or chronic conditions. Long-term use of steroid is associated with many side effects, including osteoporosis, aseptic joint necrosis, adrenal insufficiency, gastrointestinal, hepatic and ophthalmologic effects, hyperlipidemia, growth suppression, and possible congenital malformations [19]. The unfavorable effects of long-term use of steroids on bone have been known since 1932. Pathophysiological studies show that steroids affects both hematopoietic and mesenchymal derived bone cells. They reduce osteogenesis from mesenchymal stem cells (MSCs) and direct their differentiation to adipocytes, as well as decrease the maturation, lifespan, and function of osteoblasts [20]. In addition, they induce apoptosis in osteoblasts and osteocytes [21] and stimulate osteoclast activation and bone matrix degradation genes [22]. These changes eventually lead to bone loss. It is also reported that steroids cause reduction in bone hydration, bone vascularity, bone blood flow, and bone strength. Reduced bone vascularity presents clinically as avascular necrosis [23].

Although the negative effect of steroids on bone is well documented, their impact on dental implant osseointegration and implant success is not fully understood. Furthermore, most of the data comes from animal studies, case reports, and retrospective studies. Animal models with osteoporosis-like conditions induced by steroid administration showed reduced implant–bone contact, altered extracellular matrix expression, and impaired implant osseointegration [24–26]. In contrast, separate case reports showed that long-term steroid use did not affect implant osseointegration [27–29]. Case series showed similar conclusions with no contraindication for implant placement [30, 31].

In this case report, the patient was on long-term use of steroids to control her chronic disease. She had been on different stages with remission and relapse that required the use of different courses of steroids. Although the patient DXA scan shows normal bone density, the implant treatment was unsuccessful. This could indicate that steroids could affect bone metabolism and the normal healing process.

Recent systematic review has shown the important role of vitamin D in implant osseointegration in animals and humans [32]. Low vitamin D levels can cause early implant failure, and supplements improve the bone level and the outcome of implant treatment. However, the patient in this report is on calcium and vitamin D supplements to control the steroid side effects, and the levels were within normal levels according to her medical records.

We can speculate the age of the patient as an important factor in this case. This is in contrast to all other case reports and case series that reported the effect of steroid use on older patient population. The impaired implant healing and osseointegration could be attributed to steroid use, which not only lasted for a long time but also started at a very young age, which may have caused major changes in bone metabolism and affect healing capacity.

It is important to note that patient started courses of rituximab to better control relapses.Rituximab is a genetically engineered monoclonal antibody targeting CD20. It was the first monoclonal antibody to be approved for use in the treatment of cancer. The binding of rituximab to CD20 expressed on B cells leads to B cell depletion and suppression of the interaction between B cells and T cells. Since INS was primarily postulated to be a disorder in T cell function, rituximab helps to control relapses by enhancing the regulatory T cell function [33]. The effect of rituximab on implant healing and osseointegration has not been reported. Its effect on the immune system is transient and depends on the dose, frequency of infusion, and medication combination. The patient was under close follow-up during the periods of medication administration and was cleared by her physician before any dental procedure.

It is important to evaluate patients’ medical and drug history thoroughly before placing dental implants. Consultation with the patient’s physician and requesting the necessary laboratory and radiographic tests should be completed prior to any treatment. Although placing implants in steroid users is not contraindicated, it is still controversial. Each case should be evaluated individually, and the treatment should be formulated according to overall patient health. Previously proposed guidelines based on a case of implant complication in a steroid user [34] include cessation of steroid use during the healing phase of the dental implant after consultation with the patient’s physician, as well as the use of preoperative antibiotics. Clinical practice guidelines for placing dental implants in a chronic steroid user should be clearly framed.

## Data Availability

All data generated or analyzed during this study are included in this published article.

## Abbreviations

NS: Nephrotic syndrome
INS: Idiopathic nephrotic syndrome
MCD: Minimal change disease
DXA: Bone density scan
